# A review on the relationship between the distal 1q21.1 microdeletion and schizophrenia

**DOI:** 10.3389/fgene.2025.1612654

**Published:** 2025-07-28

**Authors:** Xinpeng Guo, Junrong Guo, Xijing Liu, Ting Hu

**Affiliations:** ^1^ Department of Medical Genetics, West China Second University Hospital, Sichuan University, Chengdu, Sichuan, China; ^2^ West China School of Medicine, Sichuan University, Chengdu, Sichuan, China; ^3^ Key Laboratory of Birth Defects and Related Diseases of Women and Children (Sichuan University), Ministry of Education, Chengdu, Sichuan, China

**Keywords:** schizophrenia, distal 1q21.1 microdeletion, *PRKAB2*, *BCL9*, *CHD1L*

## Abstract

Schizophrenia is a complex neuropsychiatric disorder closely associated with genetic factors. Copy number variations (CNVs) play a key role in the genetic etiology of schizophrenia, with the distal 1q21.1 microdeletion identified as a rare CNV that serves as a significant genetic risk factor for the disorder. This microdeletion is found in 0.2%–0.6% of individuals with schizophrenia and is associated with an eightfold increased risk of developing the condition. The distal 1q21.1 region contains several schizophrenia risk genes, including *PRKAB2*, *BCL9*, *CHD1L*, *GJA5*, and *GJA8*. This review focuses on the roles of these five genes in brain function and explores their potential pathophysiological mechanisms in schizophrenia. By synthesizing current evidence, this review aims to deepen the understanding of schizophrenia by outlining its genetic architecture and molecular mechanisms, thereby providing a comprehensive framework for exploring disease pathogenesis.

## 1 Introduction

Schizophrenia is a severe mental disorder characterized primarily by hallucinations, delusions, cognitive impairments, and disorganized speech and behavior (Birnbaum and Weinberger, 2017). It is a multifactorial disease with a high hereditary component, with an estimated heritability of approximately 80% (Legge et al., 2021). Schizophrenia not only affects the diagnosed individual but also presents significant challenges to families and society, affecting about 1% of the global population (Birnbaum and Weinberger, 2017; Smeland and Andreassen, 2022). Despite extensive studies, its pathogenesis remains unclear. However, recent advances in genetic research have highlighted the crucial role of genetic factors in the risk of schizophrenia. In the 1980s (Sherrington et al., 1988), first suggested a potential link between schizophrenia and a specific chromosome (chromosome 5) through familial linkage studies. The advent of genome-wide association studies (GWAS) by the early 21st century marked significant progress, enabling the identification of numerous susceptibility loci associated with the disorder (O'Donovan et al., 2008; Iyegbe and O'Reilly, 2022; Biological insights from 108 schizophrenia, 2014). These findings underscore that schizophrenia, better conceptualized as a syndrome or a spectrum rather than a single disease entity, is associated with multiple genetic loci, reflecting its biological and clinical heterogeneity.

In recent years, emerging evidence has highlighted that CNVs contribute to the increased risk of schizophrenia (Rare chromosomal deletions and duplications, 2008; Bergen et al., 2019). Numerous epidemiological studies have determined that CNVs are closely associated with the genetic etiology of schizophrenia (Mollon et al., 2023; Rees et al., 2011; Rees et al., 2016). Among the known genetic alterations in schizophrenia, CNVs are one of the most common pathogenic changes. Marshall et al. (2017) conducted a genome-wide study with 41,321 participants, including 21,094 schizophrenia cases and 20,227 controls. The study identified a significant increase in CNV burden in schizophrenia, particularly at the 1q21.1 region (OR = 1.11, P = 5.7 × 10^−15^), even after excluding previously identified risk loci. The 1q21.1 region harbors several genes involved in synaptic function and neurodevelopment. The increased CNV burden in this region may disrupt the normal function of these genes, affecting neurodevelopment and synaptic function, thereby elevating the risk of schizophrenia. Among rare CNVs, the distal 1q21.1 microdeletion has been identified as a significant genetic risk factor for schizophrenia, occurring in 0.2%–0.6% of schizophrenia cases (Marshall et al., 2017; Levinson et al., 2011; Guo et al., 1993). Furthermore, individuals with 1q21.1 microdeletions have an eightfold increased risk of schizophrenia, further highlighting the significant association between distal deletions at 1q21.1 and schizophrenia (Rare chromosomal deletions and duplications, 2008; Stefansson et al., 2008). This review summarizes the evidence linking the distal 1q21.1 microdeletion to schizophrenia and outlines the mechanisms of action of five genes (*PRKAB2, CHD1L, GJA5, GJA8, BCL9*) in the nervous system and their potential relationship with schizophrenia.

## 2 Chromosomal structure and genetic mechanisms of the 1q21.1

**FIGURE 1:**
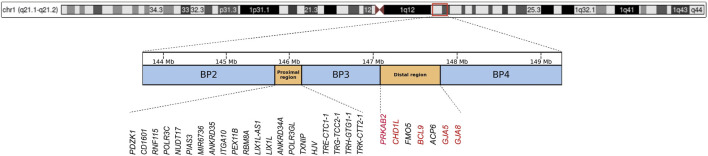
Chromosomal structure of the 1q21.1-1q21.2 region (hg38), with blue boxes representing the three breakpoint regions (BP2, BP3, and BP4), and orange boxes indicating the Proximal and Distal regions. Genes within the Proximal and Distal regions are annotated below, with schizophrenia-associated risk genes in the Distal region highlighted in red.

Chromosomal region 1q21.1 is a structurally complex genomic locus, characterized by abundance of low-copy repeats, which makes it highly prone to non-allelic homologous recombination (Malhotra and Sebat, 2012). This susceptibility leads to recurrent chromosomal deletions and duplications. The region harbors four primary segmental duplication clusters, termed breakpoints (BP1-BP4), which can be classified into two distinct regions: the proximal (BP2-BP3) and distal (BP3-BP4) regions (Brunetti-Pierri et al., 2008; Rosenfeld et al., 2012). The distal 1q21.1 microdeletion refers to a deletion of approximately 0.8 Mb between BP3 and BP4 (located at approximately 147.1–147.9 Mb, hg38). This region contains at least seven unique protein-coding genes, including *PRKAB2*, *CHD1L*, *FMO5*, *BCL9*, *ACP6*, *GJA5*, and *GJA8* (Edwards et al., 2021) (Figure 1). According to data from the UK Biobank (https://www.ukbiobank.ac.uk), the prevalence of distal 1q21.1 microdeletions in general population is approximately 0.027% (Kendall et al., 2017). Among individuals with developmental delay, intellectual disability, and/or congenital malformations, the detection rate of distal 1q21.1 microdeletions is approximately 0.2% (Guo et al., 1993). This microdeletion is usually inherited in an autosomal dominant manner, with inheritance possible from either parent. Carriers may exhibit either normal or abnormal phenotypes. Additionally, approximately 18%–35% of cases are *de novo* (Guo et al., 1993). The clinical presentation of 1q21.1 distal microdeletions shows considerable variability, with incomplete penetrance (Edwards et al., 2021; Upadhyai et al., 2020). Affected individuals may be asymptomatic or may present with a range of neurodevelopmental disorders, including developmental delay, autism spectrum disorder (ASD), schizophrenia, and attention-deficit hyperactivity disorder (Rare chromosomal deletions and duplications, 2008; Guo et al., 1993; Mefford et al., 2008; Busè et al., 2017; Bucan et al., 2009; Gudmundsson et al., 2019; Bernier et al., 2016). Furthermore, this microdeletion has been associated with other features such as cataracts, microcephaly, congenital heart defects, ocular anomalies, seizures, and renal abnormalities (Guo et al., 1993; Brunetti-Pierri et al., 2008; Mefford et al., 2008; Busè et al., 2017; Bernier et al., 2016; Christiansen et al., 2004; Costain et al., 2016; Weber et al., 2011).

## 3 Impact of distal 1q21.1 microdeletion on brain structural development

Sønderby et al. (2021) conducted a large-scale neuroimaging meta-analysis, revealing dose-dependent effects on brain structures in carriers of distal 1q21.1 microdeletions, which were accompanied by cognitive deficits. The study demonstrated that distal 1q21.1 microdeletions exert a positive dose-dependent effect on intracranial volume and total cortical surface area, particularly in the frontal and cingulate cortices, while negatively affecting the caudate nucleus and hippocampus volume. These findings link distal 1q21.1 microdeletions to structural brain changes, suggesting that this genetic variation may increase the risk of neuropsychiatric disorders by affecting structural changes during brain development. Szecówka et al. (2023) further demonstrated that individuals at high risk for schizophrenia exhibited reduced subcortical volumes, including the hippocampus and thalamus, supporting the hypothesis that volumetric changes in brain structures are associated with psychiatric disorder risk. Notably, alterations in key brain regions, particularly the hippocampus, may play a crucial role in schizophrenia pathogenesis. The hippocampus, a critical region for memory and emotional regulation, has been shown to exhibit significant structural changes in individuals with schizophrenia, including reduced hippocampal volume, which is associated with cognitive deficits and emotional dysregulation (Peterson et al., 2023; Sasabayashi et al., 2021; Wegrzyn et al., 2022). Consistent with previous study, van (van Erp et al., 2016) reported significant reductions in subcortical structure volumes in patients carrying schizophrenia-associated CNVs, including 1q21.1 microdeletions/microduplications and 22q11.2 deletion syndrome, compared to CNV carriers without schizophrenia. These findings suggest that distal 1q21.1 microdeletions not only disrupt normal brain development but also contribute to abnormal volumetric changes in specific brain regions, further emphasizing the role of distal 1q21.1 microdeletions in the risk of neuropsychiatric disorders. Additionally, Boen et al. (2024) identified region-specific effects beyond overall brain measurement differences in distal 1q21.1 microdeletions. Collectively, these findings indicate that distal 1q21.1 microdeletions contribute to schizophrenia by altering early brain structure development, thereby supporting the hypothesis that the distal 1q21.1 microdeletion is significant genetic risk factors for schizophrenia.

Induced pluripotent stem cell (iPSC) models have been utilized to examine the impact of distal 1q21.1 microdeletions on neuronal proliferation, differentiation, maturation, and synaptic function, revealing disruptions in early neurodevelopment, potentially associated with an increase in lower-layer cortical neurons. These findings were corroborated in mouse models carrying the 1q21.1 deletion (Chapman et al., 2022). In particular, (Nielsen et al., 2017) demonstrated that distal 1q21.1 microdeletions contribute to schizophrenia pathophysiology by affecting dopaminergic signaling in the Df (h1q21)/+ mouse model. The Df (h1q21)/+ mice exhibited several schizophrenia-like behaviors, including hyperactivity in response to amphetamine, impaired prepulse inhibition (PPI), and altered dopamine receptor sensitivity, which are consistent with the positive symptoms seen in human schizophrenia. Further, Gordon et al. (2021) found a general downregulation of mitochondrial-related gene expression in neurons from 1q21.1 microdeletion mice, consistent with transcriptomic alterations observed in postmortem brains of individuals with schizophrenia and ASD, suggesting neuronal energy dysfunction plays a crucial role in schizophrenia pathogenesis. Overall, the results from iPSC models, murine models, and transcriptomic analyses highlight the critical role of distal 1q21.1 microdeletions in disrupting neuronal development, synaptic function, and energy metabolism, contributing to the pathophysiology of neurodevelopmental disorders involving schizophrenia. These findings further support distal 1q21.1 microdeletions as genetic risk factors for schizophrenia and related neurodevelopmental conditions.

## 4 Risk genes within distal 1q21.1 region involving in schizophrenia

### 4.1 PRKAB2

*PRKAB2* (OMIM: 602741) encodes the β2 subunit of the AMP-activated protein kinase (AMPK) complex, a highly conserved heterotrimeric kinase critical for cellular energy metabolism and acting as an essential energy sensor within cells (Ronnett et al., 2009; Hardie et al., 2012; Hardie, 2007; Zhou et al., 2022). Specifically, in the nervous system, AMPK regulates energy metabolism, provides neuroprotection, modulates neuronal conduction, and participates in neural development, all of which are vital for maintaining normal neuronal function (Brunetti-Pierri et al., 2008; Muraleedharan and Dasgupta, 2022; Bobela et al., 2017; Shah et al., 2017; Yang et al., 2022; Belforte et al., 2021). Moreover, the functional significance of AMPK in the central nervous system is further supported by its suppressive effect on the mTOR signaling pathway, a key regulatory axis involved in synaptic plasticity, learning processes, and memory formation (Harvard et al., 2011; Wang and Jia, 2023). AMPK regulates glucose metabolism in the brain by modulating glucose uptake, glycolysis, and glycogen metabolism, thereby maintaining energy homeostasis and proper neuronal function (Muraleedharan and Dasgupta, 2022; Dienel, 2019; Muraleedharan et al., 2020). Bioenergetic dysfunction, including aberrant insulin signaling and impaired glucose homeostasis, has been implicated in schizophrenia pathophysiology, contributing to core clinical symptoms (Henkel et al., 2022; Bryll et al., 2020; Agarwal et al., 2020). In this context, evidence shows that a single copy deletion of *PRKAB2* reduces the expression of AMPK-β2, thereby impairing AMPK activation. Conversely, duplication of *PRKAB2* does not adversely affect AMPK activity (Harvard et al., 2011). Further, Nagy et al. (2018) have demonstrated that a deficiency of PRKAB2 and AMPK complex activity in the nervous system of a fruit fly model leads to decreased learning ability and severe sleep disturbances. These findings are particularly relevant because cognitive impairments and changes in sleep patterns are common symptoms of schizophrenia. Thus, *PRKAB2* may increase the risk of developing this condition. Furthermore, deficiency in AMPK can shorten lifespan and cause abnormalities in neuronal dendritic structures, a phenotype also associated with schizophrenia (Nagy et al., 2018). Interestingly, Ng et al. (2012) discovered that activation of AMPK effectively alleviates dopaminergic signaling deficits and mitochondrial dysfunction in *Drosophila* models of Parkinson’s disease. However, this protective effect is lost when AMPK is inactivated. In a comprehensive approach, (Wagh et al., 2021) through integrated analysis of various studies, identified 227 differentially expressed genes (DEGs) between schizophrenia patients and controls, demonstrating that *PRKAB2* significantly appears among these DEGs, showing genetic and epigenetic changes associated with schizophrenia. Additionally, it indicates that polymorphisms in *PRKAB2* are associated with weight gain in patients with schizophrenia or affective disorders treated with antipsychotic drugs (chlorpromazine or olanzapine) (Souza et al., 2012). Based on these findings, *PRKAB*2 plays a pivotal role in energy metabolism and the maintenance of normal function within the nervous system. Its genetic deficiency may impair AMPK activation, thereby disrupting cerebral energy metabolism and ultimately contributing to cognitive deficits and sleep disturbances, which are common symptoms associated with schizophrenia. Therefore, the haploinsufficiency of *PRKAB2* may be closely linked to the onset and progression of schizophrenia.

### 4.2 BCL9

*BCL9* (OMIM: 602597), located at 1q21.1, is a schizophrenia susceptibility gene that encodes a nuclear retention factor for β-catenin. It plays a crucial role in the Wnt signaling pathway, a key regulatory mechanism in neural development that modulates the proliferation, migration, and differentiation of neural stem cells. Additionally, this pathway is essential for maintaining neuroplasticity and promoting neurogenesis (de la Roche et al., 2008; Yoon and Mao, 2021; Adachi et al., 2004; Hoseth et al., 2018; Kuwabara et al., 2009; Zechner et al., 2003). Wnt signaling is activated during neural development and is crucial for synaptic plasticity in the adult brain (Jensen et al., 2012; He et al., 2018). The neurodevelopmental hypothesis of schizophrenia implicates disturbances in this pathway in the pathogenesis of schizophrenia (Inestrosa et al., 2012; Panaccione et al., 2013; Okerlund and Cheyette, 2011). A GWAS covering the Han Chinese population, including 5,772 controls and 4,187 schizophrenia patients, revealed multiple single nucleotide polymorphisms (SNPs) located in the *BCL9* gene that are significantly associated with schizophrenia (Li et al., 2011). Additionally, another GWAS analyzing data from 1,774 European and American schizophrenia patients and 2,726 controls identified three SNPs in the *BCL9* strongly linked to negative symptoms of schizophrenia. Among these, rs583583 showed the most significant association, further suggesting a potential role of *BCL9* in schizophrenia susceptibility (Xu et al., 2013). However, a study conducted by Kimura et al. (2015) examined the association between the rs583583 polymorphism and the manifestation of negative symptoms in Japanese individuals diagnosed with schizophrenia. Their results indicate that *BCL9* is unlikely to harbor a common genetic variant that contributes to the increased risk of schizophrenia in the Japanese population. Additional GWAS and meta-analyses provide further evidence supporting these findings, suggesting that *BCL9* is implicated in the negative symptoms of schizophrenia and designating it as one of the most prominent high-risk candidate genes for the disorder. Notably, other components of Wnt signaling have also been found to be associated with schizophrenia and other mental illnesses, further emphasizing the pathway’s significant role in schizophrenia (Yoon and Mao, 2021). Disruptions during early neurodevelopment have been closely associated with the underlying pathological mechanisms of schizophrenia (Birnbaum and Weinberger, 2017; Cheng et al., 2022; Ahmad et al., 2018; Weinberger, 2017). Wnt proteins are essential components of critical signaling pathways that regulate fetal brain development, contributing to angiogenesis, neurogenesis, cell survival, synaptic development, and neuronal extension, thereby playing indispensable roles in these processes. β-catenin, a key component of the classical Wnt pathway, whose mutations have been identified as a common cause of intellectual disability, highlights its importance in the development of the neural system (Yoon and Mao, 2021). Schizophrenia is also marked by aberrant mRNA expression of Wnt-related genes, weakening typical β-catenin-dependent signaling and enhancing atypical Wnt signaling. Schizophrenia features abnormal expression of Wnt-related genes and abnormal levels of plasma proteins, suggesting that drugs targeting the Wnt pathway may play a role in the treatment of severe mental disorders (Hoseth et al., 2018; Freyberg et al., 2010). *BCL9* has emerged as a key genetic contributor to schizophrenia susceptibility through its regulation of the Wnt pathway. Moreover, specific SNPs within the *BCL9* gene have been significantly associated with the negative symptoms of schizophrenia, further supporting its potential as a risk candidate gene.

### 4.3 CHD1L

*CHD1L* (OMIM: 613039) (Chromodomain helicase/ATPase DNA binding protein 1-like gene) encodes the *CHD1L* protein, a multifunctional factor involved in chromatin remodeling processes essential for DNA replication, transcription regulation, repair, and recombination. In addition, it exerts a crucial role in various cellular biological processes, including cell differentiation and development (Xiong et al., 2021; Wang et al., 2021; Jiang et al., 2015; Ahel et al., 2009). *CHD1L* exhibits the highest expression in the brain, where it is pivotal for nervous system development (Brockschmidt et al., 2012). A study on human embryonic stem cells (hESC) demonstrated that the overexpression of *CHD1L* upregulates the expression of ectodermal genes, particularly the key regulatory gene *PAX6*, which is critical for neural development. Such upregulation promotes the differentiation of hESCs into neuroepithelia. Conversely, the knockout of *CHD1L* significantly impairs this differentiation process (Dou et al., 2017). Neuroepithelial cells, as progenitor cells in the early nervous system, differentiate into various types of neurons and glial cells, forming the complex structure of the brain and nervous system (Merkle and Alvarez-Buylla, 2006; Eze et al., 2021). Pahlevan et al. (2024) reported that knockdown of *CHD1L* in human iPSC-derived neurons, along with its knockout in zebrafish, disrupt normal neuronal development and impair neural function. The importance of *CHD1L* in early nervous system development is further underscored by findings suggesting its central role in normal neuronal differentiation and function. Furthermore, studies indicate that disturbances in neuronal differentiation could be linked to the development of schizophrenia (Notaras et al., 2022; Robicsek et al., 2013; Robicsek et al., 2018). *CHD1L* participates in DNA damage response through interactions with multiple repair-associated factors, thereby promoting genomic stability and supporting the fidelity of DNA repair pathways (Xiong et al., 2021; Wang et al., 2021; Bulut-Karslioglu et al., 2021). Moreover, studies suggest that polymorphisms in DNA repair genes are potential etiological factors for mental disorders, and impaired DNA repair functions may increase the risk of schizophrenia (Odemis et al., 2016; Yang et al., 2017; Benes, 2011). Currently, the hypothesis that genetic damage to DNA and/or repair genes is linked to the pathogenesis of schizophrenia is supported by gradually accumulating evidence. However, the causal relationship between DNA damage/repair and schizophrenia remains controversial, highlighting that our current understanding of DNA damage and repair mechanisms in these disorders is still evolving (Markkanen et al., 2016). The relationship between *CHD1L* and schizophrenia likely involves its role in both neuronal differentiation and DNA repair. While the precise causal connection between *CHD1L* and schizophrenia remains unclear, growing evidence suggests that genetic variations or functional alterations in *CHD1L* may contribute to the pathogenesis of schizophrenia by impairing neurodevelopment and compromising genomic stability, thereby elevating the risk of the disorder.

### 4.4 GJA5, GJA8

The connexins (Cxs) encoded by *GJA5* (OMIM: 121013) and *GJA8* (OMIM: 600897) are a class of transmembrane proteins that are essential components of gap junctions, essential for intercellular communication. The Cx40 protein encoded by *GJA5* is primarily expressed in the cardiac conduction system and atria, where it facilitates electrical coupling of atrial myocytes. Similarly, the Cx50 protein, encoded by *GJA8*, is mainly expressed in the lens epithelium, where it plays a crucial role in maintaining the transparency of the human lens (Bai, 2014; Ceroni et al., 2019; Brodie et al., 2019; Dong et al., 2024). In addition, *GJA5* and *GJA8* are also expressed in the nervous system, where Cx40 and Cx50 participate in neuronal gap junctions, regulating neuronal excitability and synaptic plasticity in the central nervous system. Connexin-mediated gap junctions facilitate electrotonic coupling within chemically homogeneous GABAergic networks, allowing rapid ion exchange and promoting synchronized neuronal firing, thereby contributing to fast and spatially distributed inhibition of neural excitability (Lapato and Tiwari-Woodruff, 2018; Decrock et al., 2015; Rash et al., 2001). Moreover, the loss of astroglia gap junction functionality may lead to severe cognitive impairments in patients with schizophrenia (Mitterauer, 2009; Mitterauer, 2011). Importantly, *GJA5* and *GJA8* are considered candidate genes for schizophrenia, largely due to their involvement in neural signaling and their location within the 1q21 chromosomal region, which is consistently linked to the disorder. Studies using cumulative scoring have found that *GJA5* and *GJA8* are genes frequently disrupted by CNVs in schizophrenia. Integrating accumulated priority data with known schizophrenia susceptibility genes, further analysis has identified *GJA8* as a promising candidate gene for schizophrenia, supported by two independent pieces of evidence (from CNVs and genetic association or linkage studies) (Luo et al., 2014). In a paired case-control sample from Toronto, Canada, the Cx50 rs989192-rs4950495 haplotype was found to be associated with schizophrenia, a finding that was replicated in a Portuguese family study. Finally, analyses of alleles, genotypes, and haplotypes have not found an association between Cx40 and schizophrenia, highlighting that it is Cx50, not Cx40, that may play a role in the genetic susceptibility to schizophrenia (Ni et al., 2007). Overall, the exact mechanisms by which *GJA5* and *GJA8* contribute to schizophrenia remain unclear. However, based on existing evidence, it is hypothesized that these genes, which encode Cxs, play a critical role in intercellular communication and membrane junctions. Haploinsufficiency of *GJA5* and *GJA8* may disrupt gap junction assembly or electrical coupling, thereby affecting the transmission of information across neural circuits. This disruption of neural communication could contribute to the pathogenesis of schizophrenia by impairing the function of various neural networks.

## 5 Concluding remarks and future perspectives

Schizophrenia is a complex and multifactorial neurodevelopmental disorder, involving genetic factors. The distal 1q21.1 microdeletion is a major genetic risk factor for schizophrenia, with studies showing an eightfold increased risk in affected individuals. This review discusses the potential roles of genes within the 1q21.1 region, particularly *PRKAB2*, *BCL9*, *CHD1L*, and *GJA5/GJA8*, and their possible molecular mechanisms in the pathogenesis of schizophrenia. Supplementary Table S3 summarizes the identified risk genes and their biological roles in disease-related alterations.

The pathogenesis of schizophrenia may involve the synergistic action of multiple genes, such as *PRKAB2*, *BCL9*, *CHD1L*, *GJA5*, and *GJA8*. For example, mutations in *PRKAB2* may disrupt neuronal energy metabolism, leading to imbalances in neurotransmitter synthesis and release, which could underlie many of the neurochemical disturbances observed in schizophrenia. Mutations in *BCL9* may impair the Wnt signaling pathway, which is crucial for neuronal differentiation and the formation of neural networks. Mutations in *CHD1L* could interfere with chromatin remodeling during early neurodevelopment, impacting the expression of key genes necessary for neuronal differentiation. Moreover, as the nervous system matures, mutations in *GJA5* and *GJA8* could disrupt the connectivity and synchronization of neuronal signals, further impairing the function of neural circuits. The combined effects of these genetic alterations can ultimately lead to dysfunction in critical brain regions such as the cortex and hippocampus, which are involved in cognitive functions, perception, and memory. This results in the hallmark symptoms of schizophrenia, including cognitive impairments, hallucinations, delusions, and negative symptoms. Thus, we suppose that the integrated effects of these genetic variations form a complex network of pathogenic mechanisms that contribute to the broad clinical spectrum of schizophrenia.

Despite significant progress in current research, identifying consistent genetic markers remains challenging due to clinical heterogeneity and genetic diversity of schizophrenia. While CNVs, such as the 1q21.1 microdeletion, are implicated in schizophrenia, the precise mechanisms through which these genetic alterations contribute to the spectrum of symptoms are still unclear. Additionally, the interaction between genetic and environmental factors requires further exploration. Future research should focus on integrating multi-omics approaches, including genomics, transcriptomics, and proteomics, to better understand the molecular pathways involved in schizophrenia. Moreover, advanced animal models and iPSC technologies offer promising avenues for further investigation into the relationship between 1q21.1 and schizophrenia pathogenesis.
