# Supplementary material for: A review on the relationship between the distal 1q21.1 microdeletion and schizophrenia
Source: Front Genet. 2025 Jul 28;16:1612654. doi: 10.3389/fgene.2025.1612654 (PMC12336040; doi:10.3389/fgene.2025.1612654)
Supplement: Supplementary file 1 [file Table1.docx]

**Table 1. Schizophrenia risk genes in the distal 1q21.1 region**

| Risk Gene | Full Name | Function | Gene Expression Deficiency and Its Effects | Mechanisms Underlying Schizophrenia Risk |
| --- | --- | --- | --- | --- |
| PRKAB2 | Protein kinase AMP-activated non-catalytic subunit beta 2 | Encodes the β2 subunit of AMPK complex, which plays a critical role in cellular energy metabolism | Downregulation of AMPK-β2 expression, resulting in impaired AMPK activation | 1.Deficiency in AMPK complex activity impairs learning abilities and causes severe sleep disturbances  2.Deficiency in AMPK leads to abnormal dendritic structure in neurons |
| BCL9 | BCL9 transcription coactivator | Encodes the nuclear retention factor β-catenin, which plays a critical role in the regulation of the Wnt signaling pathway | Inhibits the Wnt signaling pathway, potentially affecting neuronal differentiation, migration, and proliferation, and interfering with synaptic plasticity | 1.Disruption of the Wnt signaling pathway increases the risk of schizophrenia  2.Associated with negative symptoms of schizophrenia |
| CHD1L | Chromodomain helicase DNA binding protein 1 like | Encodes the CHD1L protein, which is involved in chromatin remodeling, transcriptional regulation, cell differentiation, development, as well as DNA repair and recombination | 1.Leads to impaired differentiation of neuroepithelial cells, resulting in neurodevelopmental defects  2.Impairs DNA repair function, affecting genomic stability | 1.Impaired neurodevelopment increases the risk of schizophrenia  2.Disruption of genomic stability increases the risk of schizophrenia |
| GJA5/ GJA8 | Gap junction protein alpha 5/ Gap junction protein alpha 8 | Encodes a connexin (Cxs), a fundamental component of gap junctions, primarily involved in intercellular communication and signal transduction | Affects the transmission of neural electrical signals, leading to dysregulation of neural circuits | Disruption of neural circuits is associated with the pathogenesis of schizophrenia |
